# Lightwave-electronic harmonic frequency mixing

**DOI:** 10.1126/sciadv.adq0642

**Published:** 2024-08-14

**Authors:** Matthew Yeung, Lu-Ting Chou, Marco Turchetti, Felix Ritzkowsky, Karl K. Berggren, Philip D. Keathley

**Affiliations:** ^1^ Research Laboratory of Electronics, Massachusetts Institute of Technology, 77 Massachusetts Ave., Cambridge, MA 02139, USA.; ^2^ Institute of Biophotonics, National Yang Ming Chiao Tung University, Linong Street, Beitou District, Taipei City 112304, Taiwan.

## Abstract

Electronic frequency mixers are fundamental building blocks of electronic systems. Harmonic frequency mixing in particular enables broadband electromagnetic signal analysis across octaves of spectrum using a single local oscillator. However, conventional harmonic frequency mixers do not operate beyond hundreds of gigahertz to a few terahertz. If extended to the petahertz scale in a compact and scalable form, harmonic mixers would enable field-resolved optical signal analysis spanning octaves of spectra in a monolithic device without the need for frequency conversion using nonlinear crystals. Here, we demonstrate lightwave-electronic harmonic frequency mixing beyond 0.350 PHz using plasmonic nanoantennas. We demonstrate that the mixing process enables complete, field-resolved detection of spectral content far outside that of the local oscillator, greatly extending the range of detectable frequencies compared to conventional heterodyning techniques. Our work has important implications for applications where optical signals of interest exhibit coherent femtosecond-scale dynamics spanning multiple harmonics.

## Teaser

Compact petahertz-scale electronic frequency mixers enable broadband field-resolved optical detection.

## INTRODUCTION

**Fig. 1.**
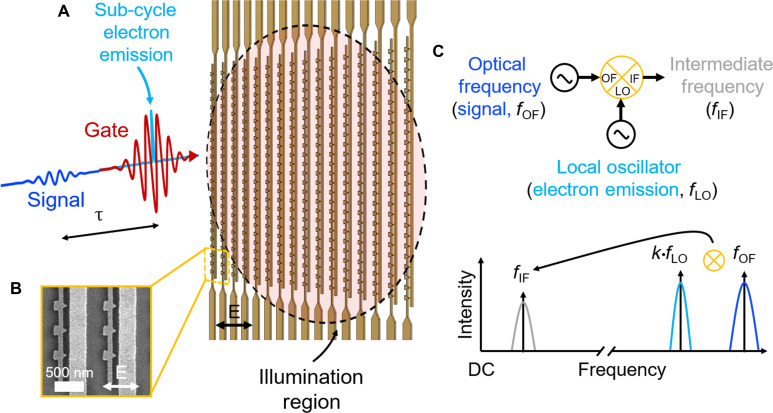
Schematic of experimental measurement. (**A**) A gate pulse illuminates the nanoantenna network and drives sub-optical cycle electron emission. A small signal is introduced over a variable delay. This small signal modulates the electron emission from the nanoantennas leading to the optical-frequency mixing process. Both the gate and signal pulse polarization are parallel to the nanoantenna tip axis as indicated by the black arrow. (**B**) A representative scanning electron microscope image showing the nanoantennas and the polarization incident on the nanoantennas. (**C**) The devices can be conceptualized as electronic harmonic frequency mixers (top schematic) with the gate waveform of central frequency *f*_gate_ serving as the local oscillator (LO, with central frequency *f*_LO_ = *f*_gate_), and the signal having central frequency *f*_signal_ as the optical frequency input (OF, with central frequency *f*_OF_ = *f*_signal_). The mixing process (bottom schematic) provides a current signal at baseband [intermediate frequency (IF)] for the detection of harmonics of the local oscillator *kf*_LO_ (right plot). Here, we measure the baseband response for field-resolved measurement of the signal as a function of delay τ.

Lightwave electronics [also often called petahertz (PHz) electronics] seek to integrate optics and electronics effectively, leveraging sub-cycle information contained within the ultrafast oscillations of light fields (*1*–*5*). In this pursuit of electronics operating at optical frequencies, a substantial obstacle arises from the mismatch between the characteristic frequencies of optical (PHz regime) and conventional electronic systems for readout (gigahertz to terahertz). To solve similar issues in frequency mismatch in more conventional radio-frequency electronics, nonlinear frequency mixers are used, with a myriad of applications including radar, cellular phone service, and radio communications. Harmonic frequency mixers in particular enable the use of a single local oscillator to capture information from both the fundamental and higher-order harmonic frequency channels, bringing their information content down to lower, baseband frequencies for readout (*6*) (see Fig. 1C). Compact PHz-electronic harmonic frequency mixers would enable field-resolved optical signal analysis spanning octaves of the optical spectrum within a single device. Here, we demonstrate the use of plasmonic nanoantennas as lightwave-electronic harmonic frequency mixers (see Fig. 1) for the field-resolved characterization of harmonic optical waveforms (PHz scale).

To provide a more flexible, field-resolved readout of optical signals using electronic systems, early efforts in the 1970s aimed to extend electronic harmonic frequency mixing techniques to mid-infrared frequencies (up to 88 THz) using metal point-contact diodes (*6*). However, progress stagnated until recent advancements in optical and nanofabrication technologies. Recent work has shown that nanoscale needle tips and plasmonic antennas having nanoscale vacuum channels act as nonlinear electronic diode elements similar to the earlier point-contact diodes (*6*, *7*). Through their carrier-envelope phase (CEP) sensitivity (*8*–*13*), use in field sampling (*14*, *15*), and measurements of their photoemission response (*16*–*20*), researchers have demonstrated that their electronic response can extend up to 1 PHz and beyond.

While past work has demonstrated the use of sub-cycle, optical-field emission from nanoantennas and needle tips for field-resolved optical waveform characterization (*14*, *15*), these demonstrations were limited to CEP-stable few-cycle waveforms and frequencies contained within the spectrum of the gate waveform driving the electron emission. Here, we experimentally demonstrate how multicycle, non–CEP-stable sources can be used for field-resolved analysis extending to signal waveforms with central frequencies well higher than that of the driving gate waveform through harmonic frequency mixing. Specifically, we show that the highly nonlinear, broadband electronic response of plasmonic nanoantennas enables lightwave-electronic harmonic frequency mixing into the PHz regime for optical signal processing.

In our proof-of-concept measurement, we use electrically connected nanoantenna devices for accurate amplitude and phase-resolved readout of both the fundamental (0.177 PHz, 1690 nm) and second harmonic (0.353 PHz, 850 nm) fields of an optical waveform using only the fundamental waveform as the local oscillator (see Fig. 1 for an overview). Our measurements demonstrate how, under multicycle operation, PHz-electronic nanoantennas can be conceptualized and used as harmonic frequency mixers to greatly extend the bandwidth of time-domain, field-resolved optical detection beyond one octave of spectral coverage without the need for prior nonlinear conversion in crystals, spectral phase retrieval, single-cycle waveform generation, or CEP stabilization. Our study highlights a crucial connection between lightwave electronics and traditional nonlinear electronics. This connection serves to unite the electronics and optical physics communities, filling a gap in existing literature predominantly focused on strong-field and optical physics. By clarifying this link, this work acts as a bridge between these two fields.

The increased bandwidth obtained through harmonic mixing enables seamless amplitude- and phase-resolved characterization of nonlinear processes of interest, such as solid-state harmonic generation (*21*–*30*), coherent Raman scattering (*31*, *32*), and multiphoton processes (*33*–*36*), without the need for nonlinear frequency conversion, spectral phase retrieval, or a spectrally overlapped local oscillator reference. In the far term, we anticipate that lightwave-electronic harmonic mixer devices will provide basic building blocks for field-resolved electromagnetic signal detection and processing at optical frequencies.

### PHz harmonic mixing for optical waveform analysis

Recent work has shown that cross-correlation using the nonlinear photoemission from gases and nanostructures as the electronic readout enables field-resolved optical waveform characterization with sub-cycle resolution (*14*, *15*, *37*–*46*). Each technique starts with a strong gate waveform that drives sub-cycle photoelectron emission (red curve in Fig. 1A). A weak signal (blue curve in Fig. 1A) with the same polarization as the gate pulse then perturbs the photoemission response [perturbative method, see, e.g., (*15*, *41*, *47*, *48*)] or is cross-polarized and shifts the electron momentum [streaking-like method, see, e.g., (*44*, *45*, *49*, *50*)] as a function of delay τ. The streaking-like method results in a time-integrated interaction over the signal field, resulting in a delay-dependent photocurrent that is proportional to the signal’s vector potential. For the perturbative method, however, the delay-dependent current relates directly to the signal’s electric field through an instantaneous coupling between the signal and gate waveforms analogous to the coupling of voltage waveforms in nonlinear electronic frequency mixers. Here, we focus on the perturbative method.

In this section, we first briefly introduce how these perturbative, nonlinear cross-correlation measurements provide amplitude and phase information of the signal. Our treatment focuses on the field-driven photoemission response from asymmetric nanoantenna structures like those used in our experiment but could be extended to other systems. Following this introduction, we show how these perturbative cross-correlation measurements can be viewed through the lens of nonlinear electronic frequency mixing. This framing allows us to better understand how nonlinear, field-driven photoelectron emission devices provide field-resolved readout across spectral harmonics using only a single local oscillator without the need for CEP stabilization. It also provides a framework for understanding how we might translate technologies used now at lower frequencies (e.g., radio frequency or microwave) into the PHz regime.

**Fig. 2.**
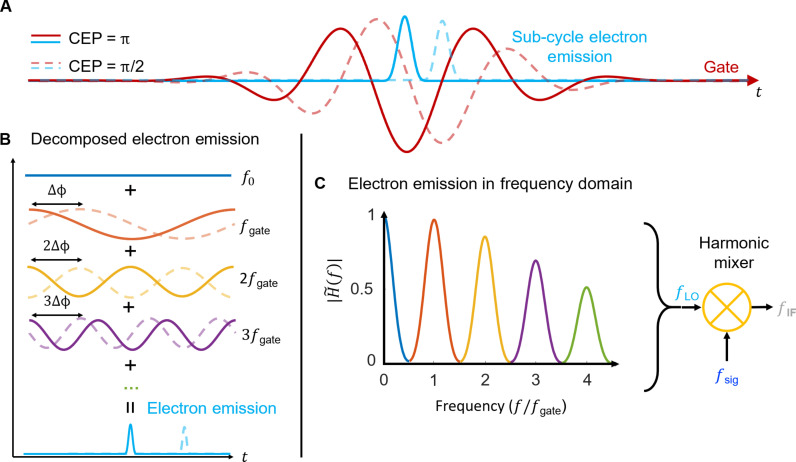
How sub-cycle emission enables harmonic frequency mixing. (**A**) Depiction of sub-cycle electron emission calculated using the FN tunneling rate (teal) driven by a single-cycle pulse for a CEP = π and CEP = π/2 (dashed). (**B**) The sub-cycle electron emission comprises integer harmonic frequencies of the gate frequency *f*_gate_, collectively contributing to the sub-cycle electron emission. At each of these frequencies, a phase shift occurs when the CEP of *f*_gate_ is altered by Δϕ (here, π/2). Specifically, for the fundamental frequency *f*_gate_, the phase shift corresponds to Δϕ, while the second harmonic corresponds to 2 × Δϕ, the third harmonic to 3 × Δϕ, and subsequent higher harmonics to *k* × Δϕ; *k* is the harmonic order. (**C**) The calculated transfer function amplitude $|\tilde{H}\left(f\right)|$ for a four-cycle Gaussian pulse with a center frequency of 0.177 PHz.

When driven by intense, few-cycle waveforms polarized parallel to the tip axis, sub-optical cycle tunneling current (*8*, *9*) can be emitted from a nanoantenna (connected triangle features in Fig. 1, A and B) to a collector (adjacent wires in Fig. 1, A and B). This configuration leads to a half-wave rectified current response relative to the driving field at the antenna apex. In Fig. 2A, we show a calculation of the sub-cycle electron emission (blue) as a function of a few-cycle gate field (red) for two different values of CEP (solid versus dashed lines). Note that we have used shorter gate pulse durations in Fig. 2 for illustration purposes. The current emission was modeled using the Fowler-Nordheim (FN) rate equation as described in (*10*–*12*, *14*, *15*, *51*) (see further discussion in section S2.1).

A nonlinear cross-correlation measurement, similar to that discussed in (*15*, *41*, *43*), can be modeled by using a strong gate waveform, in addition to a weak signal waveform (to be measured). When the signal and gate are superimposed with the same polarization and the relative delay τ between the two is varied, the time-averaged current across the nanoantenna gap can be modeled as

$$I\left(\tau \right)\propto \int_{-T_{\text{rep}}/2}^{T_{\text{rep}}/2}‍\Gamma \left[E_{\text{gate}}\left(t-\tau \right)+E_{\text{signal}}\left(t\right)\right]\;\mathrm{dt}$$ (1)

where Γ is the FN equation *T*_rep _the repetition rate, *E*_signal_ the electric field of the signal waveform, and *E*_gate_ the electric field of the gate waveform. Given that *E*_signal_ is sufficiently small, the electric field perturbation seen by the nanoantenna can be assumed to be linear and *E*_gate_ can be Taylor-expanded to the first order. The resulting current is then approximated by

$$I\left(\tau \right)\propto \int_{-T_{\text{rep}}/2}^{T_{\text{rep}}/2}‍\Gamma \left[E_{\text{gate}}\left(t-\tau \right)\right]+\left[{\left.\frac{d\Gamma }{\mathrm{dE}}\right|}_{E_{\text{gate}}\left(t-\tau \right)}\cdot E_{\text{signal}}\left(t\right)\right]\;\mathrm{dt}$$ (2)

In this equation, the integral of the second term corresponds to the measured electric field waveform. This integral represents the small-signal cross-correlation between ${\frac{d\Gamma }{\mathrm{dE}}|}_{E_{\text{gate}}\left(t-\tau \right)}$ and *E*_signal_(*t*), which we denote as *I*_cc_(τ). Because this expression represents a cross-correlation, in general, the Fourier-transformed expression can be written as ${\tilde{I}}_{\text{cc}}\left(\omega \right)\propto ℱ{\left[\frac{d\Gamma }{\mathrm{dE}}{|}_{E_{\text{gate}}}\right]}^{*}\cdot {\tilde{E}}_{\text{signal}}\left(\omega \right)$, where $\tilde{H}\left(\omega \right)\propto {\tilde{I}}_{\text{cc}}\left(\omega \right)/{\tilde{E}}_{\text{signal}}\left(\omega \right)=ℱ{\left[\frac{d\Gamma }{\mathrm{dE}}{|}_{E_{\text{gate}}}\right]}^{*}$ is the full complex frequency response of the output relative to the signal field, which is plotted as a function of frequency in Fig. 2C.

Note that in Fig. 2C, we see that the detector frequency response contains frequency components at harmonic orders both higher and lower than that of the central frequency of the gate. The operating principle behind this is identical to that of a nonlinear electronic harmonic frequency mixer where the gate generates the local oscillator with *f*_LO_ = *f*_gate_, *f*_LO_ being the local oscillator frequency and *f*_gate_ being the central frequency of the gate waveform. Because of the high nonlinearity of the FN tunneling rate Γ and half-wave rectification, the current response contains frequencies outside of the optical local oscillator centered at every integer harmonic (*14*). This is visualized in Fig. 2B where we show how the sub-cycle burst in charge over one period can be expressed as a sum of harmonic frequency components. These electronic frequency components effectively serve as frequency-distributed local oscillators that mix with the small signal. The different frequency components then provide the baseband response for amplitude and phase-resolved readout.

Conceptualizing the devices as electronic optical frequency mixers aids in describing important properties of the devices. First, it becomes apparent that CEP locking of the gate and signal pulse is not a requirement for amplitude and phase-resolved waveform readout even for the case of signals composed of higher-order harmonics provided that the gate and signal exhibit relative phase locking. Consider the case of a perfectly sinusoidal gate (i.e., local oscillator) and signal functions, where the signal is a harmonic of the gate. Let the signal then be a harmonic of the gate frequency *f*_sig_ = *kf*_gate_ as represented in Fig. 1C. We can then represent ${\frac{d\Gamma }{\mathrm{dE}}|}_{E_{\text{gate}}\left(t-\tau \right)}$ as an expanded series of harmonics of the gate frequency

$${\left.\frac{d\Gamma }{\mathrm{dE}}\right|}_{E_{\text{gate}}\left(t-\tau \right)}=h_{0}+\frac{1}{2}\left(\sum_{n=1}^{\infty }‍{\tilde{h}}_{n}e^{\text{in}\varphi }e^{i2\pi nf_{\text{gate}}\left(t-\tau \right)}+c.c.\right)$$ (3)

where φ represents the absolute phase shift of the gate, analogous to the CEP for the case of a pulsed gate. Likewise, the signal is represented by

$$E_{\text{signal}}\left(t\right)=\frac{1}{2}{\tilde{a}}_{k}e^{\mathrm{ik}\varphi +i\Delta \varphi }e^{i2\pi kf_{\text{gate}}t}+c.c.$$ (4)

where Δφ represents any remaining phase difference introduced by the measurement apparatus in addition to *k*φ. The DC output response is then formed by the multiplication of conjugate and nonconjugate coefficients of ${\tilde{h}}_{k}$ and ${\tilde{a}}_{k}$ and is found to be

$$I_{\text{cc}}\left(\tau \right)\propto \frac{1}{4}{\tilde{h}}_{k}^{*}{\tilde{a}}_{k}e^{i2\pi kf_{\text{gate}}\tau }e^{i\Delta \varphi }+c.c.$$ (5)

Note that because the signal and current responses are both phase-locked to the local oscillator (gate), the absolute phase terms φ always cancel and the harmonic mixing response is not sensitive to any fluctuations of the absolute phase φ.

These behaviors translate directly to the case of pulsed gate and signal inputs. A finite envelope of the gate pulse leads to a broadening of the harmonic pass bands as shown in Fig. 2C. These shifts are equal and opposite to those of optically generated harmonic pulses meaning that as long as the source of harmonics and the nonlinear optical radiation exhibit relative phase locking, the sampled response does not depend on the absolute CEP. We note here that absolute CEP information is effectively lost for a multi-cycle gate and a CEP-unstable laser. However, using a near single-cycle gate with a CEP-stable laser would allow one to extend this field-resolved measurement to a sampling measurement where the true electric field is measured, rather than a measurement of an ensemble of CEP values.

In addition to highlighting the role of CEP dependence on the readout, we see the importance of high nonlinearities and rectification in extending device bandwidth across harmonics. While one can certainly obtain a mixing response via conventional heterodyning and homodyning using an *E*^2^ detector (e.g., a photodiode) (*52*), we see from this analysis that an *E*^2^ current response without rectification would not yield higher harmonic components in the electronic response. However, higher harmonic terms appear at every integer harmonic for higher-order nonlinearities and half-wave rectification. For the plasmonic detectors in this work, these nonlinearities can exceed *E*^10^.

## RESULTS AND DISCUSSION

### Experimental demonstration

In this section, we present experimental results demonstrating PHz harmonic mixing through field-resolved optical waveform characterization. First, we performed degenerate waveform characterization where we used a 10-cycle near-infrared gate and signal waveforms both having a central frequency of 0.177 PHz, corresponding to when *k* = 1 in Eq. 4. This measurement illustrates the capability of multicycle sampling without the need for CEP stabilization. We next present the characterization of the second harmonic waveform (0.353 PHz) using the fundamental (10-cycle 0.177 PHz) as the gate to demonstrate harmonic frequency mixing, corresponding to *k* = 2 in Eq. 4.

**Fig. 3.**
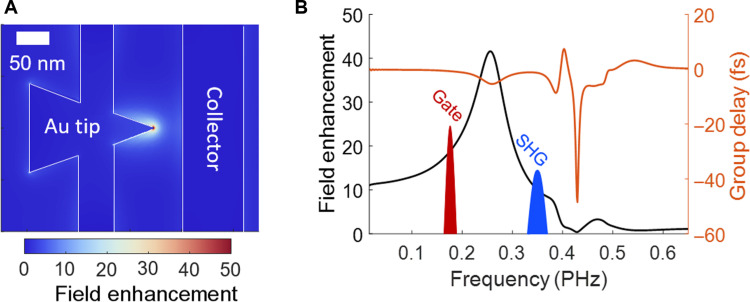
Nanoantenna design. (**A**) Finite-difference time-domain (FDTD) simulation of the electric field enhancement at the tip of a gold nanoantenna. (**B**) FDTD simulation of the field enhancement and group delay imparted by the antenna response as a function of frequency. Within the spectrum, we highlight the experimental frequencies used with the gate at frequency 0.177 PHz and a higher frequency signal at 0.353 PHz, which corresponds to the second harmonic of the gate (SHG).

In our measurements, we used an asymmetric nanoantenna design as shown in Fig. 3A as this naturally breaks inversion symmetry and enables frequency response [ $\tilde{H}\left(f\right)$ ] to have both even and odd integer harmonics (*53*). Gold was chosen as the antenna material and fused silica as the substrate. In Fig. 3B we plot the frequency-dependent field enhancement (black curve) and group delay (red curve) at the antenna apex relative to the incident light for our chosen design. The spectral regions covered by the fundamental (shaded red) and its second harmonic (shaded blue) are also shown for reference. The nominal nanoantenna geometry was chosen to have a resonant frequency between that of the fundamental and second harmonic, corresponding to a triangle base width of 180 nm and a height of 240 nm. This choice was for two reasons. First, it results in a compromise where the fundamental and second harmonic both have maximal field enhancement (≈15× and ≈10×, respectively). Second, because the fundamental and second harmonic both excite the antennas off-resonance, they experience a negligible amount of intensity reshaping and group delay dispersion (less than 2.5-fs change in group delay over the bandwidth of the fundamental and second harmonic), meaning that the excited field waveforms at the antenna apex are not noticeably reshaped in time relative to the incident field waveforms. Furthermore, this demonstrates that operating the devices on-resonance is not necessary for the practical use of optical waveform analysis. For more details regarding device design and fabrication, see the Materials and Methods.

To perform the degenerate waveform characterization, we used a 10-cycle (57 fs) 0.177-PHz (1690 nm) pulse from a commercial optical parametric amplifier (LightConversion Cronus 3P) for both the gate and the signal. This measurement differs from the work of Bionta *et al.* (*14*), which uses the on-resonance property of the nanoantennas to perform degenerate waveform characterization using a 2.5-cycle pulse. The gate and signal pulses were prepared using a Mach-Zehnder interferometer (MZI), and the pulse duration was confirmed through frequency-resolved optical gating (FROG) measurements [see fig. S5 for the measurement schematic and fig. S6 for FROG (section S2.2)].

**Fig. 4.**
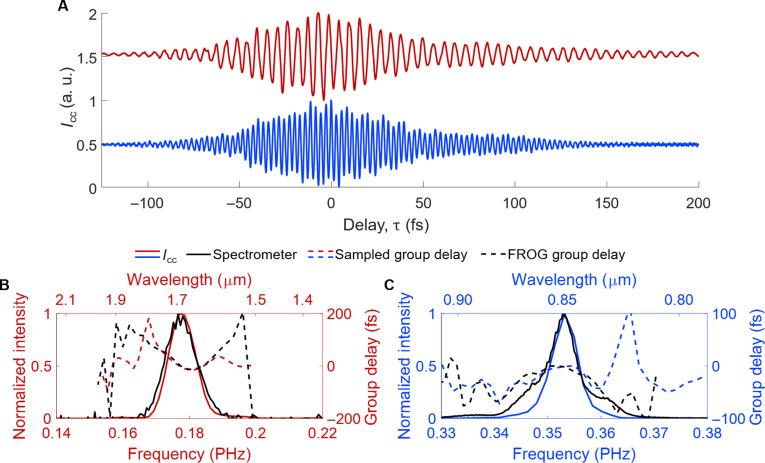
Harmonic frequency mixing for field-resolved characterization. (**A**) The measured electric field of the 10-cycle, 0.177-PHz fundamental (top, red) and measured electric field of the 0.353-PHz second harmonic (bottom, blue). The same 10-cycle, 0.177-PHz pulse was used as the gate (local oscillator) for both cases. (**B**) The corresponding frequency-domain intensity of the measured electric field (solid red line) compared to a commercial spectrometer (black solid line). The extracted group delay from the measured field (dashed red line) is compared to the group delay retrieved from a FROG measurement (dashed black line). (**C**) The corresponding frequency-domain intensity of the measured second harmonic electric field (solid blue line) compared to a commercial spectrometer (black solid line). The extracted group delay from the sampled optical field (dashed blue line) is compared to the group delay retrieved from a FROG measurement (dashed black line).

Before performing the cross-correlation measurements, we first confirmed that the devices were operated in the optical-field emission regime through the analysis of current scaling with intensity by measuring the output current as a function of the incident gate pulse energy and verifying that the emission could be well-described through a quasi-static FN tunneling rate (see fig. S4 and section S2.1 for further details). Having confirmed operation in the optical-field emission regime, we then illuminated the antennas with a gate pulse with an energy of 5.4 nJ (0.85 V/nm) and a small signal with an energy of 4.4 pJ (24 V/μm) to obtain *I*_cc_(τ) (red curve in Fig. 4). In the experiment, the signal arm was chopped and measured using lock-in detection to isolate *I*_cc_(τ) from the constant background current. The small signal gain from the high nonlinearity of the optical-field emission response enabled the sampling of signal pulse energies on the order of 1000× smaller than those of the gate. The measured optical period was 5.6 fs, which matched the expected value for a frequency of 0.177 PHz. The pulse of the sampled field was 57 fs full width at half maximum (FWHM), in close agreement with the 58-fs FWHM pulse duration retrieved FROG measurement (see fig. S6C).

Spectral intensity and group delay analysis are shown in Fig. 4B. We find good agreement between ${|{\tilde{I}}_{\text{cc}}\left(f\right)|}^{2}$ (solid red line) and the spectral intensity as measured using a commercial grating-based indium gallium arsenide spectrometer (solid black line) after normalization. In addition, the extracted group delay of ${\tilde{I}}_{\text{cc}}\left(f\right)$ (dashed red line) is shown to be concave up and agrees well with the group delay retrieved from the FROG measurement (dashed black line). These results further confirm that *I*_cc_ accurately represents the signal field of the fundamental to within a constant phase offset.

We then performed the nondegenerate waveform characterization of the second harmonic signal waveform via harmonic mixing using the same 10-cycle gate as before (*f*_gate_ = 0.177 PHz) with the signal being the second harmonic of the gate waveform (*f*_sig_ = 0.353 PHz). We note that this measurement is different from other studies using both the first and second harmonic (ω − 2ω). The work of Arai *et al.* (*54*) uses ω − 2ω for optical phase measurements, whereas Dienstbier *et al.* (*19*) use the ω − 2ω to break symmetry for the application of ultrafast switching. In this measurement, we use a sufficiently weak second harmonic waveform to demonstrate field-resolved waveform characterization.

The second harmonic was generated by adding lenses and a type 1 beta-barium borate (β-BaB_2_O_4_, BBO) crystal to the signal arm of the MZI (see fig. S8 for measurement schematic). We illuminated the antennas on the device using a pulse energy of 4.9 nJ (0.80 V/nm) for the gate and a pulse energy of 23 pJ (49 V/μm) for the second harmonic signal (after removal of the residual fundamental through filtering). The measurement of *I*_cc_(τ) using the second harmonic signal is shown in Fig. 4A (blue curve). The measured optical period was 2.8 fs matching the expected optical period for a center frequency of 0.353 PHz.

To verify the accuracy of the sampled field, we again performed FROG and used a commercial grating-based silicon spectrometer as a reference in the same way as in the degenerate case. The pulse duration from our FROG measurement was found to be 49 fs FWHM, which matches almost perfectly with the duration of *I*_cc_(τ), being 48 fs FWHM [see fig. S9 for a comparison of FROG and *I*_cc_(τ)]. In Fig. 4C, we find that ${|{\tilde{I}}_{\text{cc}}\left(f\right)|}^{2}$ (solid blue line) is in general agreement with the spectral intensity measured using the silicon spectrometer (solid black line) after normalization. There is only a discrepancy in the intensity of the spectral wings. However, the group delay of ${\tilde{I}}_{\text{cc}}\left(f\right)$ (solid red curve) is concave down and matches relatively well with the FROG-retrieved group delay (dashed red curve). Together, these results lead us to conclude that *I*_cc_(τ) accurately represents the second harmonic signal’s field waveform to within a constant phase offset.

Several aspects of this measurement are important to emphasize. First, given that the second harmonic generation used to generate the signal was phase-locked to the second harmonic of the sampling response as noted in the Introduction, there was no need for CEP stabilization of the gate. Second, the nonlinearity of the mixing process enabled the phase-resolved, interferometric pulse readout without the need for nonlinear optical generation of a spectrally overlapped local oscillator as would typically be required for all-optical homodyning or heterodyning techniques using a standard *E*^2^ detector (*52*, *55*–*58*). The third aspect is that the direct cross-correlation outputs accurately represented the fundamental and signal waveforms as confirmed using FROG without the need for any postanalysis, such as phase retrieval, or any change in the detector setup aside from the change in the signal waveform. Last, we emphasize that for the case of the second harmonic measurement, the signal pulse was appreciably shorter than the gate (48-fs FWHM signal duration versus 60-fs FWHM gate pulse duration) and of a substantially higher carrier frequency. While techniques such as electro-optic sampling can also provide field information from nonspectrally overlapped signals, the signal frequency must be lower than that of the gate, with gate pulse envelopes shorter than the cycle time of the signal (*59*–*62*).

### Concluding remarks and outlook

Here, we used nonresonant nanoantenna networks to demonstrate a broadband, on-chip electronic optical frequency harmonic mixer using optical-field-driven tunneling. We showed how the harmonic frequency mixing process enables accurate field-resolved readout of optical signal waveforms spanning more than one octave of bandwidth using a commercial laser without the need for single-cycle pulse generation or CEP locking. If these devices were produced at scale, calibrations similar to those required for commercial spectrometers would be necessary. These calibrations, based on electromagnetic simulations, would ensure accurate field-resolved measurement across the entire device bandwidth.

In comparison to wave mixing in crystals, the optical field–driven tunneling mechanism provides access to higher-order nonlinearities, and thus larger mixing bandwidths, while eliminating the need for phase-matching or a separate photodetection element (*55*, *63*). Furthermore, unlike FROG and other spectral characterization methods, the measurements provided direct amplitude and phase information in the time domain and did not require broadband spectral measurements or phase-retrieval algorithms. While techniques such as electro-optic sampling can provide similar time-domain information, they require CEP stabilization and gate pulse envelopes shorter than the cycle time of the signal (*59*).

While we demonstrated harmonic frequency mixing through pulse characterization, we believe that similar devices will be used to create compact and sensitive optical oscilloscopes with bandwidths spanning multiple octaves. We anticipate that such optical field oscilloscopes will provide needed time-domain detection tools that will enable new approaches to the field-resolved investigation of ultrafast light-matter interactions and help accelerate the development of ultrafast source technologies (e.g., compact frequency combs and optical waveform synthesizers). Beyond time-domain, field-resolved detection, PHz-electronic mixers could also be incorporated as fundamental components within future lightwave electronic systems for PHz-scale communication and computation. Aside from conversion to baseband, they could be used to generate new sum and difference frequency signals to be routed to other nearby on-chip devices.

## MATERIALS AND METHODS

### Nanofabrication

We started with 1 cm × 1 cm fused silica pieces (MTI Corp.) and cleaned them using piranha for 10 min before use. For the nanoantenna array fabrication, we spin-coated polymethyl methacrylate A2 (Microchem) at 2750 revolutions per minute (rpm) and baked at 180°C for 2 min. Afterward, DisCharge H2O X2 (DisChem Inc.) was spun at 3000 rpm so that charging did not occur during the electron beam lithography writing process. The electron beam lithography was performed at 125 keV with a dose ranging from 4000 to 6000 μC/cm^2^ with proximity effect correction. Development of the exposed polymethyl methacrylate samples was done at 0°C in a solution of 3:1 2-propanol to methyl isobutyl ketone for 50 s. Electron beam evaporation (Temescal FC2000) was performed at 2×10^−6^ torr where we first deposited a 2-nm titanium adhesion layer and then 20 nm of gold. Liftoff was performed by submerging the samples in a 65°C solution of *N*-methyl pyrrolidone (Microchem) for 1 hour.

Contacts were made to the nanoantenna using photolithography. We spin coat nLOF 2035 at 3000 rpm, then bake the resist at 110°C for 90 s. The exposure was performed using a maskless aligner with a wavelength of 375 nm and at a dose of 300 mJ/cm^2^. After exposure, we did a postexposure bake at 110°C for 90 s, then developed for 90 s in AZ726. We then used electron beam lithography to deposit 10 nm of a titanium adhesion layer and 50 nm of gold. Liftoff was performed at room temperature in a solution of acetone for at least 6 hours. The samples were ashed for 30 s, then mounted on a printed circuit board (PCB), and wire bonded.

### Laser and measurement methods

We used a LightConversion optical parametric amplifier pumped by a Yb:KGW laser pulse picked to 1 MHz (Cronus 3P) for our experiments. The idler output was compressed using a prism pair. The samples on the PCB were used as-is in ambient conditions and the output was connected to a trans-impedance amplifier with a gain of 1 V/nA (FEMTO). We measured the signal pulse-induced current (*I*_cc_) through a lock-in measurement referenced by chopping the signal arm at ∼277 Hz. The x and y channels of the lock-in are output to an oscilloscope (Keysight) with a sampling rate of at least 25 to 50 kSa/s. Before sampling measurements, we illuminated the device with the gate pulse and ensured that the photocurrent remained constant for at least several minutes. To temporally control the delay between the signal and gate pulse, we placed the gate pulse on a closed-loop piezo stage (Piezosystem Jena) with ±14 nm (0.05 fs) repeatability. The scan time for each trace was set to 20 s and averaged accordingly. We continuously scan the delay stage in a certain direction with constant velocity to reduce measurement variation.

In every measurement, we used neutral density (ND) filters to control the power of the signal and gate pulses. After recombining the two pulses, we placed a linear film polarizer with an extinction ratio of ≥10^5^ (across all wavelengths) to ensure that the two beams were horizontally polarized. Once collinearity was ensured between the gate and the signal arm, both the signal and gate pulses were focused onto the nanoantenna chip through a reflective objective (Ealing). See fig. S5 for the schematic.

For frequency doubling, we used a type 1, 1.5-mm-thick BBO crystal with θ = 24 and ϕ =90 to generate the second harmonic (SHG) of *f*_LO_ = 0.177 PHz. The SHG (0.353 PHz) is filtered using a 0.207-PHz (1450 nm) high-frequency pass filter with ND 2 at 0.177 PHz along with a broadband achromatic half-waveplate used to rotate the SHG polarization from vertical to horizontal, to match the gate pulse. Note that the integration time was crucial for accurate measurement of the higher frequencies.

For the nondegenerate supercontinuum measurement using a 10-cycle *f*_LO_ = 0.177 PHz, we ensured that the phase induced by the optics was equivalent to when the degenerately sampled supercontinuum was measured. We also use the same device for degenerate and nondegenerate sampling.

For FROG measurements, we used the Retrieved-Amplitude N-grid Algorithmic (RANA) approach [see (*64*)] as a robust retrieval algorithm. The MATLAB code is available on the Trebino Group website. We used a 128 × 128 grid for the FROG reconstruction.

We padded the retrieved waveforms with zeros before taking a fast Fourier transform (FFT) to improve spectral resolution. This was justified as the spacing between consecutive pulses in time in the experiment is much larger (1 μs) than the time window of the retrieved waveforms.

To calculate the bare field strengths, we took the pulse energy and converted it to field strength using the corresponding intensity and a Gaussian beam approximation in time/space. For the fundamental beam, the beam spot size corresponds to 12.8 μm × 9.54 μm at the focus (FWHM). For the second harmonic, the beam spot size corresponds to 12.1 μm × 19.1 μm at the focus.

### Electromagnetic, sampling, and supercontinuum generation simulations

For the field enhancement simulations, we used the open-source finite-difference time-domain (FDTD) package PyMeep (*65*). For the gold nanoantenna and silicon oxide substrate, we used the standard materials library included in the Python package. For setting up the FDTD conditions, we used periodic boundary conditions in the nanoantenna on a silicon oxide plane with perfectly matched layers in the direction of propagation of the plane-wave source to prevent multi-reflections affecting the simulation. To obtain realistic field enhancements, we ensured that the apex of the triangle had a radius of curvature of 10 nm, which we extracted through scanning electron microscopy of our devices.

In our sampling simulations, the FN equation, expressed as Γ = αϕ^−1^*E*^2^ exp (−βϕ^3/2^/*E*), was used. Here, α is 1.54×10^−6^ A eV V^−2^, β is 6.83 eV^−3/2^V nm^−1^, ϕ represents the work function (taken as 5.1 eV), and *E* denotes the electric field. With this equation, we numerically calculated the current cross-correlation by

$$I_{\text{cc}}\left(\tau \right)\propto \int ‍\Gamma \left[E_{\text{gate}}\left(t-\tau \right)+E_{\text{signal}}\left(t\right)\right]\;\mathrm{dt}$$

using a signal-to-gate ratio of 0.03.

## Supplementary Materials

### This PDF file includes:

Supplementary Text Supplementary Experimental Information Figs. S1 to S11 References
