# Supplementary material for: Lightwave-electronic harmonic frequency mixing
Source: Sci Adv. 2024 Aug 14;10(33):eadq0642. doi: 10.1126/sciadv.adq0642 (PMC11800882; doi:10.1126/sciadv.adq0642)
Supplement: Supplementary file 1 — Supplementary Text Supplementary Experimental Information Figs. S1 to S11 References [file sciadv.adq0642_sm.pdf]

Science Advances
AAAS

Supplementary Materials for

Lightwave-electronic harmonic frequency mixing

Matthew Yeung *et al.*

Corresponding author: Matthew Yeung, myeung@mit.edu; Philip D. Keathley, pdkeat2@mit.edu

Sci. Adv. **10**, eadq0642 (2024)
DOI: 10.1126/sciadv.adq0642

The PDF file includes:

Supplementary Text
Supplementary Experimental Information
Figs. S1 to S11
References

Other Supplementary Material for this manuscript includes the following:

Data S1

1 Supplementary Text

Shown in Fig. S1a are two 10-cycle Gaussian pulses used as f_{gate}, one with a carrier frequency corresponding to 0.353 PHz and another with 0.177 PHz. The corresponding Fowler-Nordheim electron emission rate for the two Gaussian pulses is shown in Fig. S1b. Since there are several cycles, there are several bursts of electrons that are generated. The inset displays the main burst at t=0, which is modulated by the signal pulse, enabling small signal sampling. The current burst full-width at half maximum (FWHM) determines the time resolution and is 0.28 fs for f_{gate} = 0.353 PHz and 0.55 fs for f_{gate} = 0.177 PHz. Lastly, the frequency response given the 10-cycle f_{gate} is shown in Fig. S1c. Since we are using a 10-cycle pulse, there are multiple electron bursts in time, resulting in a reduction in the frequency bandwidth response. If we were using a single-cycle gate pulse, the frequency domain response would be the envelope shown in the black dashed lines.

Shown in Fig. S2a is the gate-dependent electron emission in time. The electron emission can be seen as the multiplication of a comb in time with the peaks separated depending on the device geometry. With an asymmetric design, the spacing is the optical period of the gate frequency. Mathematically, it can be described as the following:

$$\begin{gathered} h(t) = f(t) \cdot g(t) \\ k(t) = h(t) \times i(t) \end{gathered} \tag{6}$$

In time, an electronic comb (f) is generated and the amplitude of the comb follows an envelope function (g), resulting in h and is purely dependent on the gate-dependent localized electric fields at the tip. Then, h is convolved with the electron emission rate envelope with finite bandwidth (i) which is directly related to the optical period of the gate frequency.

Shown in Fig. S2b is the electron emission rate for 3 different field enhancements. When the field enhancement is small, the main electron emission burst centered at $t = 0$ plays the largest role in perturbative sampling. As the field enhancement increases by a factor of 10 (equivalently an intensity enhancement of 1000), the side electron emission bursts start to increase in amplitude and the smaller amplitude sub-optical-cycle oscillations in the electric field start to contribute to electron emission.

1.1 Phase information from sampling

In phase retrieval techniques like FROG, the sign of the phase is not determined. With harmonic frequency mixing, we have relative phase information. When we measure our time-domain trace, we directly take our data and FFT into the frequency domain and take the derivative of the phase. To demonstrate that we have the sign of the phase, we perform sampling simulations using a 1690 nm 2-cycle pulse as the signal and gate. The gate pulse has 0 fs^2 GDD and we chirp the signal pulse with + and - 200 fs^2 GDD. The corresponding field we measure in time is different depending on the GDD in the pulse Fig. S3a. In the frequency domain, the sign of the GD is resolved as shown in Fig. S3b.

2 Supplementary Experimental Information

In this section we provide supplementary experimental information important to our work.

2.1 Electron Emission Regime

There are numerous papers demonstrating that such nanoantenna devices operate in the optical field emission regime using 10s to 100s of pJ pulse energies with few-cycle pulses (*8–11, 14*). To confirm our devices are operating in the Fowler-Nordheim regime with the 10-cycle f_{LO}= 0.177 PHz gate pulse, we measured the current output versus peak field shown in Fig. S4 with a typical device that has never been used for waveform measurements, denoted as "New Device", and a device which we used for more than 4 hours of illumination (denoted as "Burned-in device"). We fit the low-pulse-energy section of the new device current vs peak field curve to the conventional multiphoton photoemission form, $I_{MP} \propto |F_0|^{2N}$, where F_0 is the incident peak field, finding a fit of N = 7 . However, we note that there is a sharp turnover of the emission which is not fully understood.

While space charge might cause such a sharp turn-over in the emission rate, we ruled this out after considering the emitted charge densities. Considering the close to $100\,\mathrm{pA}$ currents and the repetition rate of $1\,\mathrm{MHz}$ distributed over 306 antennas, we were generating 2 electrons per pulse per antenna. Given the length of the optical pulse of approximately 10 cycles, the electrons were very unlikely to interact within the same optical half-cycle. Furthermore, from Coulomb's law, we can calculate typical forces that would result in an electron $5\,\mathrm{nm}$ away from the tip in an electric field strength of $\approx\ 0.06\,\mathrm{V\,nm^{-1}}$, substantially lower than local fields of around $14\,\mathrm{V}$.

An alternative explanation is that this is emission from a surface state due to an adsorbed molecule. This is consistent with our observation that after some time of operation, we observe that the needed energy for similar emission rates increases indicated by the shift to the right of the "Burned-in Device" (red dots in Fig. S4). Our interpretation is that during the process of using the devices, surface adsorbates are removed and the emission approaches expected from Fowler-Nordheim tunneling from gold as noted in past works. In studying similar devices using

field-emission from the application of DC biases, it was shown that similar behavior is due largely to water adsorption on the surface that is removed through UV radiation (*66*). Here we feel this removal is due to the optical illumination and interaction at the tip from the gate.

We fitted the Fowler-Nordheim curve to the ”Burned-in Device” data (green curve in Fig. S4) to demonstrate that the emission is indeed well-described by quasi-static tunneling. We used the initial field of the optical waveform before enhancement in comparison with the field from this fit to determine the enhancement factor. The measured photocurrent and nonlinearity after long-term operation were sufficient to continue using the device for harmonic mixing and waveform detection. Our measurements presented in the main text operated at $\geq$0.8 V/nm, which corresponds to a Keldysh parameter of $\gamma \leq$0.6, indicating that we were operating in the tunneling regime.

Regarding device degradation, we note that previous work from Yang *et al.* ((*12*)) observed that modifying the antenna tips shifts its resonance and reduces peak field enhancement. This was attributed to reshaping due to heating from time-averaged power. In this study, we operated off-resonance and at lower repetition rates, significantly reducing average power density (by more than one order of magnitude). Consequently, we did not observe significant signal degradation over time after device ”burn-in,” as mentioned earlier.

2.2 Degenerate Waveform Analysis

For degenerate waveform analysis of 0.177 PHz (1690 nm) as shown in SI. Fig. S5, we used an MZI to generate a pulse pair. We used ND filters to attenuate the gate and signal pulses and placed a chopper in the signal arm as a reference for the measurement of the current-induced change when the small signal perturbed the sub-optical cycle gate. After the pulse pair was recombined at the second beamsplitter, we used a linear polarizer with extinction coefficients greater than 10^5 at 1690 nm.

We performed frequency-resolved optical gating (FROG) measurements of the pulses in time to provide a reference to compare against the measured fields. The error for the reconstruction shown is 5.510^{-3}. In Fig. S6a, b we show the measured and retrieved spectrograms of the 10-cycle 1690 nm pulse used in the measurements. The degenerate sampled fields in Fig. S6c show relatively good agreement in the time domain with the FROG result. In both traces a small side lobe is present in time near 65 fs. The retrieved pulse duration was 58 fs FWHM and the sampled pulse duration was 57 fs FWHM. Lastly, the frequency domain comparison between the sampled intensity, retrieved intensity, and the grating-based spectrometer is shown in Fig. S6d. The sampled group delay and retrieved group delay show very good agreement.

We also performed degenerate waveform analysis using a 63-cycle pulse with a center frequency of 0.291 PHz (1030 nm) using an air-cooled 6-watt LightConversion Carbide after characterizing the pulse using SHG FROG. To begin our measurement, we employed the built-in pulse picker to reduce the repetition rate down to 500 kHz from 1 MHz. We then directed the output of the laser through the MZI with a closed-loop linear piezo stage delay line (Smaract SLC2445-S with MCS2) with $\pm$ 40 nm ($\pm$ 0.13 fs) repeatability. Utilizing a pristine device, we measured the current as a function of the gate pulse energy. We proceeded with measurements once the total photocurrent reached approximately 20 pA (blue line in Fig. S7a), which is equivalent to 1.4 nJ (0.328 V/nm) for this specific device measured. After measurements, we remeasured the current as a function of the gate pulse energy and observed degradation (orange curve Fig. S7a) associated with long pulse durations and highly localized fields at the nanoantenna tips. We tried to obtain 20 pA of current after measuring, however, this specific device rapidly degraded at 2.3 nJ. Using a signal pulse energy of 0.02 nJ (38.6 V/um), we degenerately sampled the pulse as shown in Fig. S7b.

In the top panel (1.76 ps x-axis range) of Fig. S7b, at $\pm$ 500 fs, small side lobes are seen. As we continue zooming in until the 10 fs x-axis range, the measured optical period is 3.4 fs

and the expected optical period for a center frequency of 291 PHz is 3.4 fs. The technique can be generalized beyond 10-cycle pulses, however, since the devices were not operating in the tunneling regime for these measurements, these results have been placed in the supplemental information and require future studies to better understand the mechanism.

To verify the sampled field, we compared our measured results with SHG FROG. As seen in Fig. S7c, d is the measured and retrieved spectrograms of the 63-cycle 1030 nm pulse used in the measurements. The degenerate sampled fields in Fig. S7e show relatively good agreement in the time domain with the FROG result demonstrating that there is a small side lobe in time at $\pm$500 fs. The retrieved pulse duration is 218 fs and the sampled pulse duration is 216 fs. Lastly, shown in Fig. S7f is the frequency domain comparison between the sampled intensity, retrieved intensity, and the grating-based spectrometer. The sampled group delay and retrieved group delay are also shown and show reasonable agreement.

2.3 Non-degenerate Waveform Analysis

For non-degenerate waveform analysis of 0.353 PHz (850 nm) using a 0.177 PHz (1690 nm) gate, we modified the previously shown setup by adding a telescope for the 1690 nm gate to control the beam spot size before focusing on the nanoantennas. This modification was performed since when the SHG was generated and collimated using the type-1 1.5 mm thick BBO the beam spot was smaller in size before entering the objective. We also used a shortpass filter which was ND2 at wavelengths longer than 1450 nm as well as an achromatic half waveplate to rotate the SHG such that its polarization matched the gate pulse polarization. After the second beamsplitter, the pulse pair passed through a linear polarizer which had an extinction ratio that is $> 10^5$ at 1690 nm and $> 10^6$ at 850 nm.

We performed FROG measurements on the SHG, and the measured and retrieved spectrograms of the SHG of the 10-cycle 1690 nm pulse used in the measurements are shown in Fig.

S9a, b. The error for the reconstruction shown is 1.310^{-2}. The non-degenerate sampled fields in Fig. S9c show relatively good agreement in the time domain with the FROG result. The retrieved pulse duration was 49 fs FWHM and the sampled pulse duration was 48 fs FWHM. Lastly, shown in Fig. S9d is the frequency domain comparison between the sampled intensity, retrieved intensity, and the grating-based spectrometer. The sampled group delay and retrieved group delay also show very good agreement.

To demonstrate non-degenerate waveform analysis of a lower frequency using a higher frequency and to show that lower frequencies that are not integer harmonics can still be sampled, we used a separate few-cycle supercontinuum source with details that can be found in (*67*). After supercontinuum generation, an SF10 prism pair was used to spatially disperse the beam. After the prism pair, the Er pump was spatially filtered, and the soliton and dispersive wave contributions were spatially separated. The higher-frequency dispersive wave portion of the supercontinuum was returned through the prism pair for compression and the removal of spatial chirp and was used as the gate. We note that the lower-frequency soliton, which was used as the signal, was not returned through the prism pair leaving some residual spatial chirp.

For the gate pulse, 19 pJ (0.21 V/nm at focus) of the 3-cycle (12 fs) dispersive wave (0.250 PHz, 1200 nm) was used. For the signal, 30 pJ (0.15 V/nm at focus) of the 11-cycle (65 fs) soliton (0.166 PHz, 1600 nm) was used. The measurement configuration is shown in detail in Fig. S10a. Degenerate waveform analysis of the dispersive wave was performed and is shown in Fig. S10b. Next, we performed non-degenerate waveform analysis measurement of the soliton and measured an optical period of approximately 6.0 fs as expected as Fig. S10c. Lastly, the normalized Fourier-transformed time-domain sampled waveforms are shown in Fig. S10d. This emphasizes the ability of the nonlinear mixing process to also sample lower frequency contributions at non-integer harmonics in the few-cycle regime without CEP locking.

2.4 Regarding Alternative Materials

As these devices are based on resonant nanoantennas, we performed simulations using platinum, a metal that is not plasmonic, to demonstrate other nanoantenna materials could be used. It can be observed that toward DC frequencies (Fig. S11a), the field enhancement converges. It can be easily seen in Fig. S11b, where in the long wavelength field enhancement of the gold and platinum antennas converge, which is a characteristic of metal nanoantennas.

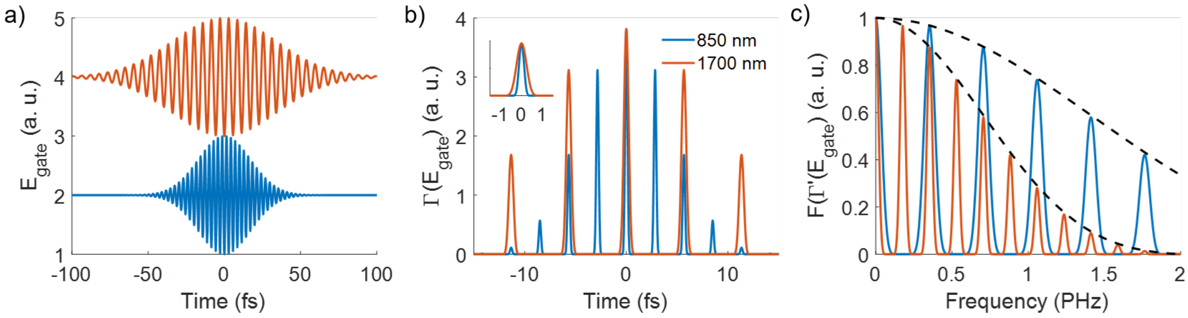

Figure S1: **The transfer function response as a function of gate frequency.** We use a 10-cycle Gaussian pulse (a) with a carrier frequency of 0.353 PHz and 0.177 PHz to illustrate the frequency bandwidth dependence when the gate pulse changes. (b) The corresponding time domain electron burst for the two gate wavelengths used. (c) The frequency response of the electron burst for the two gate wavelengths used and their corresponding envelope if a 1-cycle pulse was used (dashed lines)

3 Data and code

We have provided the original data and selected code used in this manuscript.

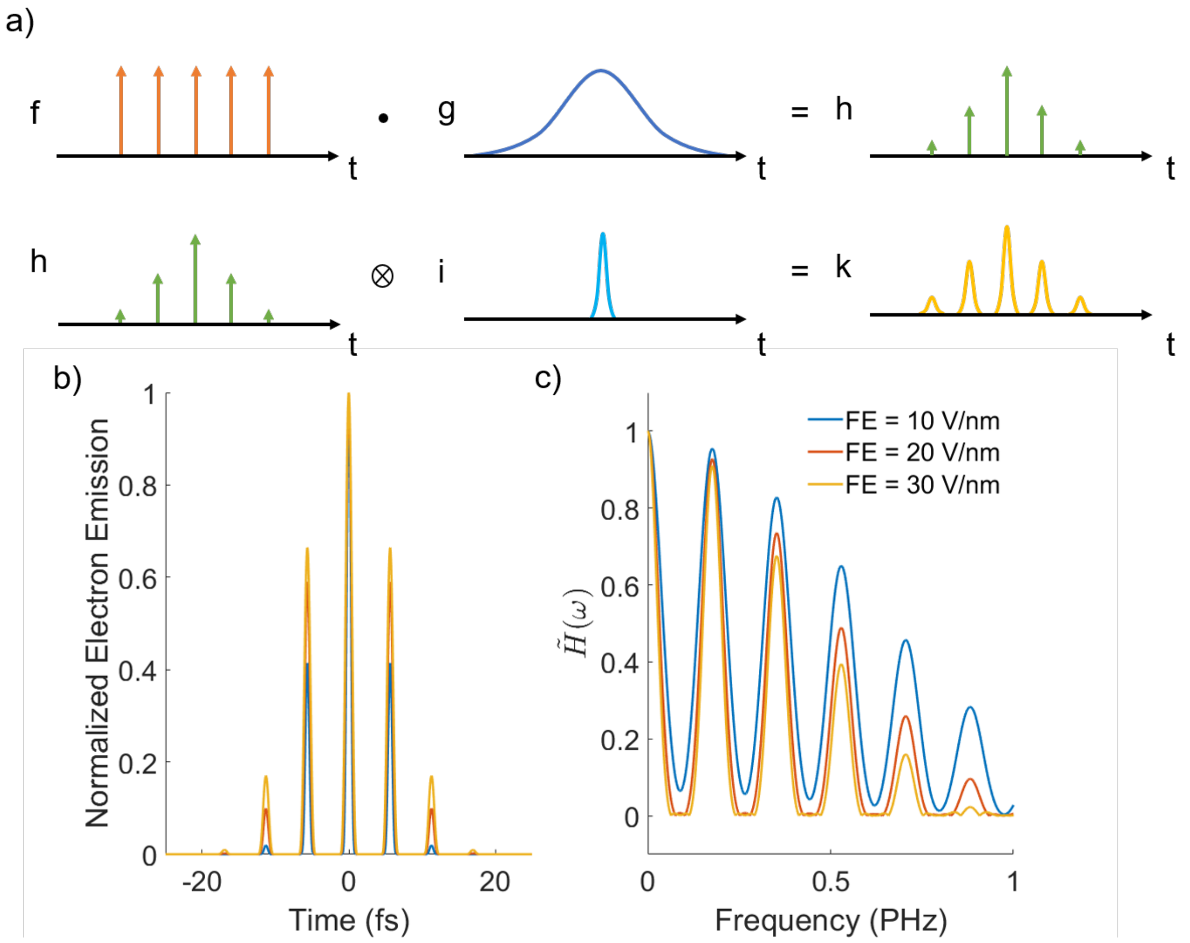

Figure S2: **Illustration of comb generation in time and how the comb is affected by field enhancement.** (a) Illustration on the electronic comb generation in time. (b) The effect of the electronic comb with varied field enhancement (FE) using a 5-cycle f_{gate} = 0.177 PHz optical field. The corresponding sub-optical-cycle electron emission burst is shown in (b) and the corresponding Fourier transform is shown in (c) as the field enhancement is increased.

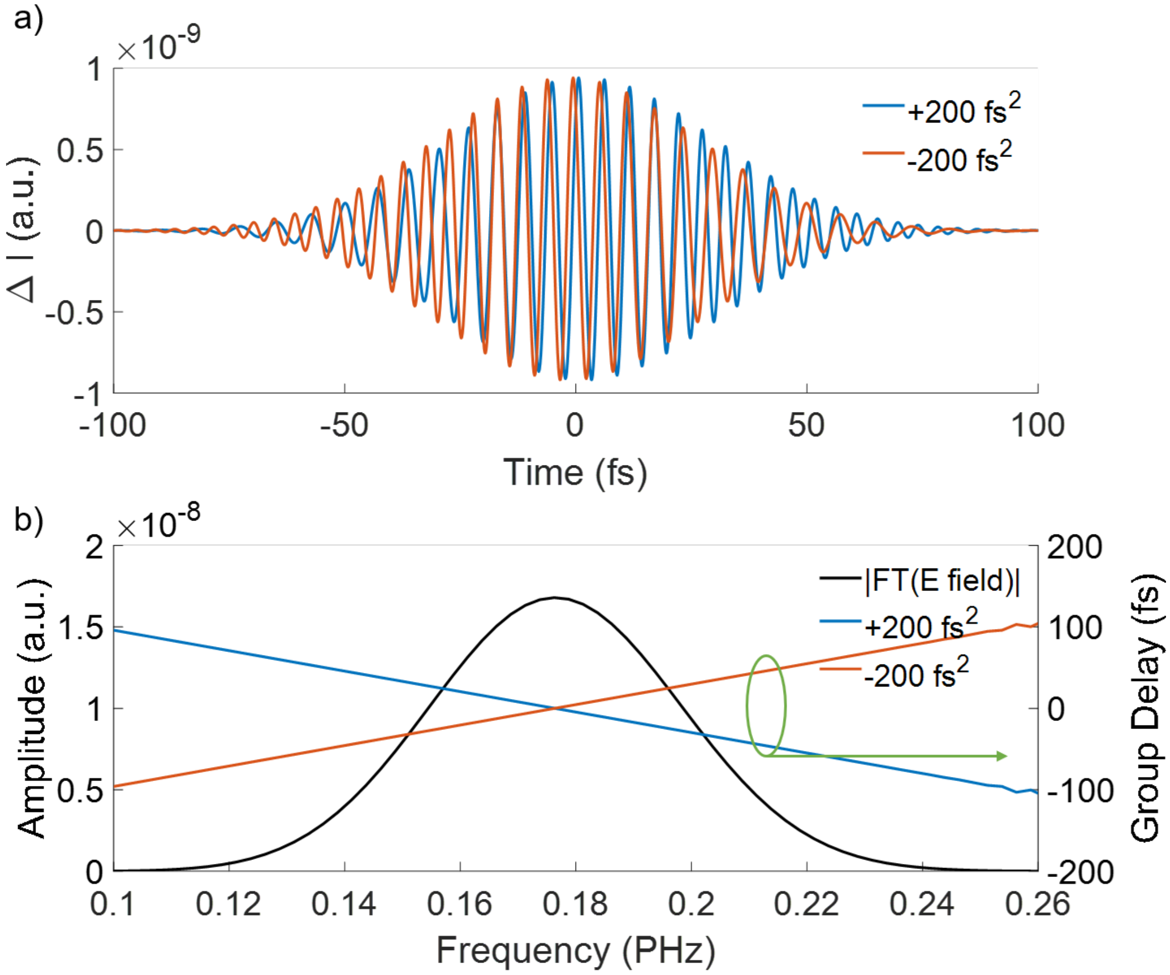

Figure S3: **Simulated sampling with a non-zero GDD signal pulse.** (a) 2-cycle 1690 nm signal pulse with + 200 fs^2 (blue) and - 200 fs^2 (orange) GDD sampled using a 2-cycle 1690 nm gate pulse. (b) The corresponding Fourier-transformed sampled fields in (a) is shown as the black line while the group delays are shown in blue and orange.

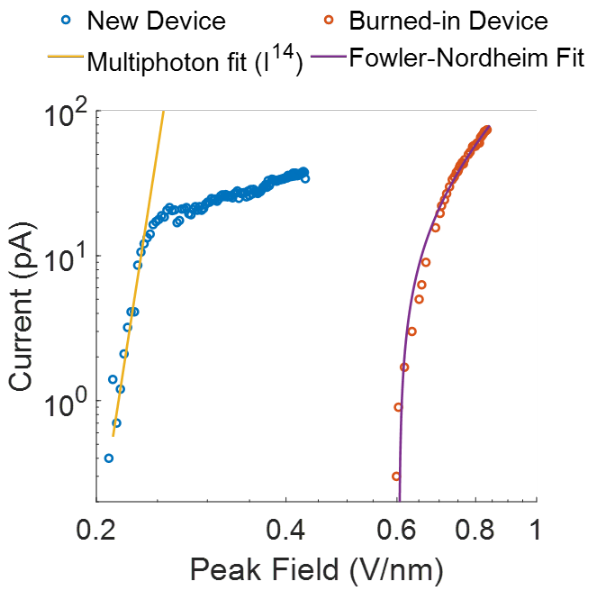

Figure S4: **Current output of the gold nanoantenna network.** The curves include a new device and the same device after performing measurements for >4 hours using the 10-cycle f = 0.177 PHz.

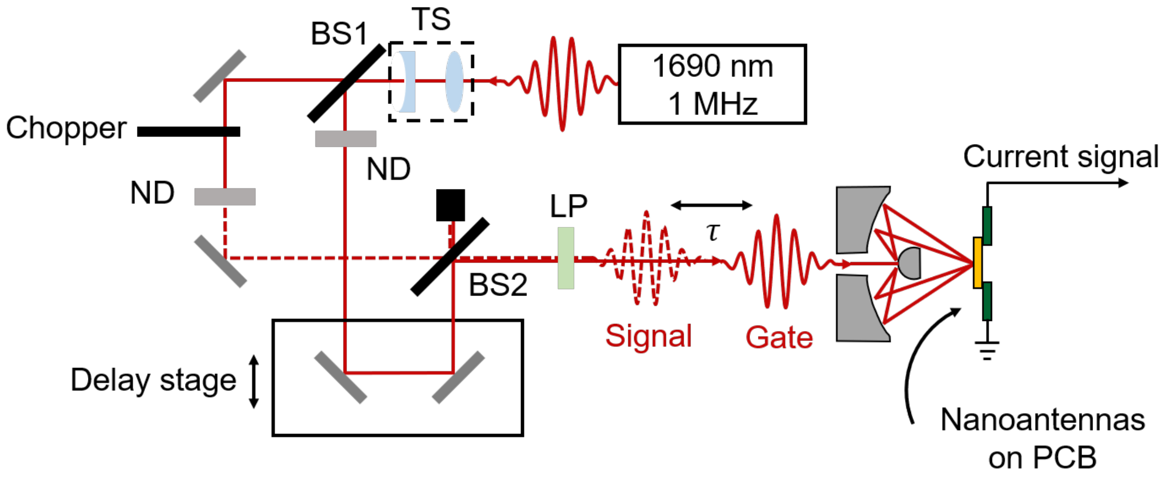

Figure S5: **A schematic of the experimental setup for the measurement of f = 0.177 PHz.** The laser light was split using a beamsplitter (BS). One arm has a delay stage and was used as the gate pulse, while the signal arm was chopped and neutral density filters were used to attenuate the signal. Eventually, the two pulses are recombined using an identical beamsplitter before being sent to a reflective objective where they are focused onto the nanoantenna devices.

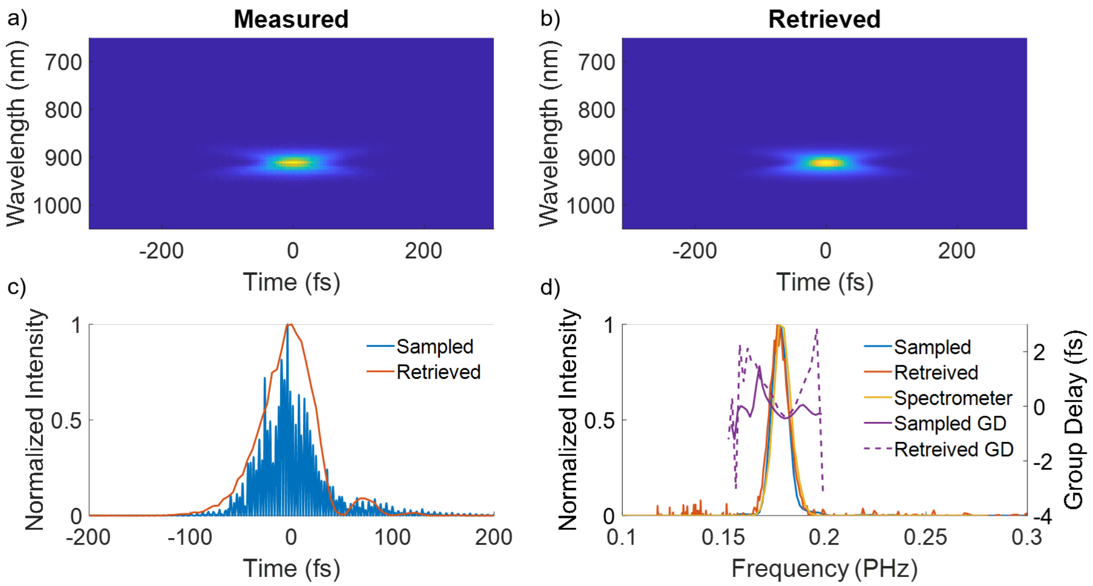

Figure S6: **Frequency-resolved optical gating (FROG) of the $f = 0.177$ PHz pulse.** (a) measured and (b) retrieved spectrograms. (c) A comparison of the squared modulus of sampled optical fields and the retrieved pulse versus time. (d) A comparison of the squared modulus of the Fourier transformed sampled fields, retrieved pulse versus frequency, a spectrometer reference, group delay from the sampled optical fields, and the retrieved group delay.

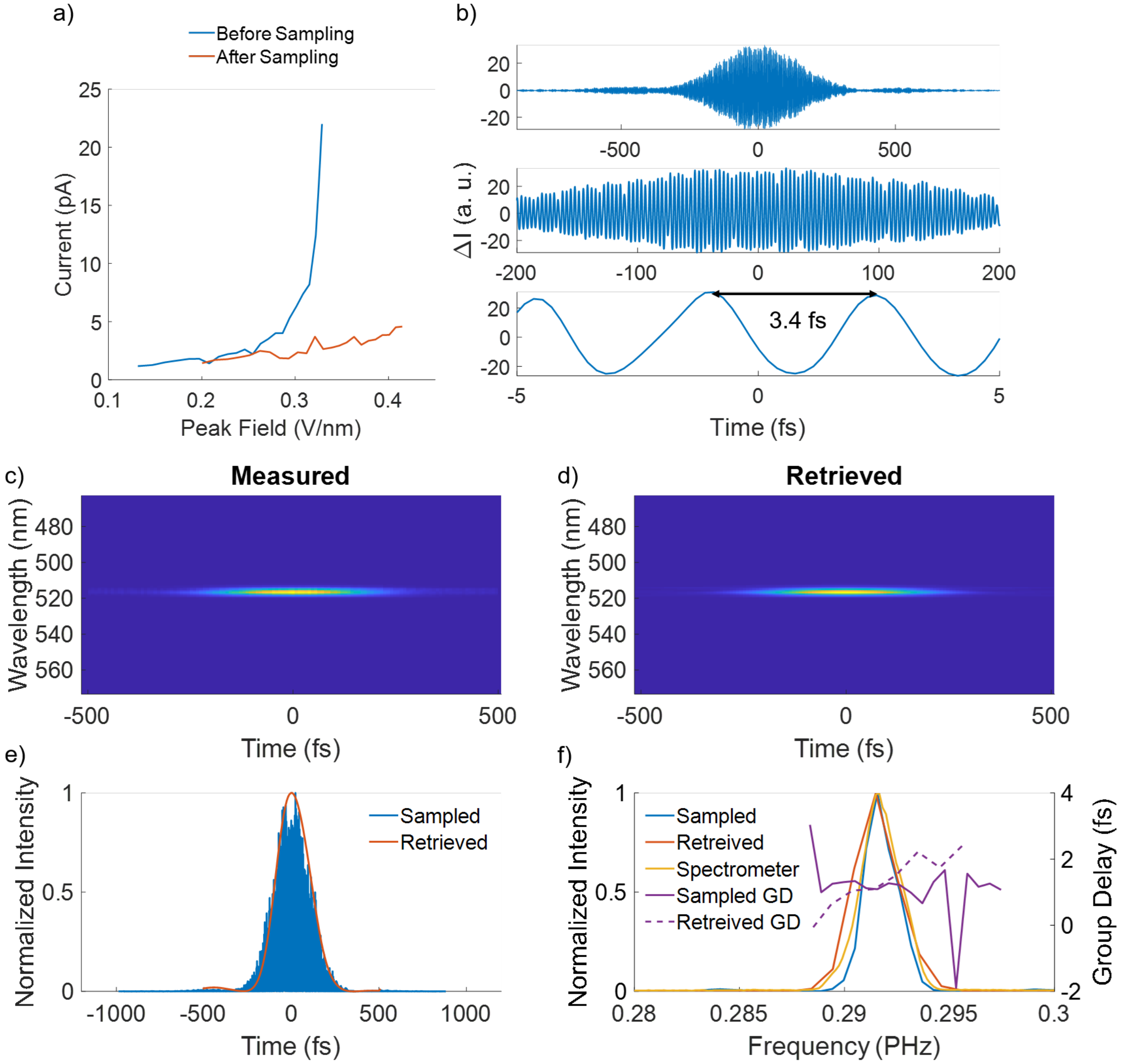

Figure S7: **Degenerate optical field sampling of a 63-cycle (218 fs) 0.291 PHz pulse.** (a) The corresponding current vs peak field before and after s ampling. (b) The degenerately sampled field with varying x-axis limits of 1760 fs, 400 fs, and 10 f s. The frequency-resolved optical gating (FROG) (c) measured and (d) retrieved spectrograms. (e) A comparison of the squared modulus of sampled optical fields and the retrieved pulse versus time. (f) A comparison of the squared modulus of the Fourier transformed sampled fields, retrieved pulse versus frequency, a spectrometer reference, group delay from the sampled optical fields, and the retrieved group delay.

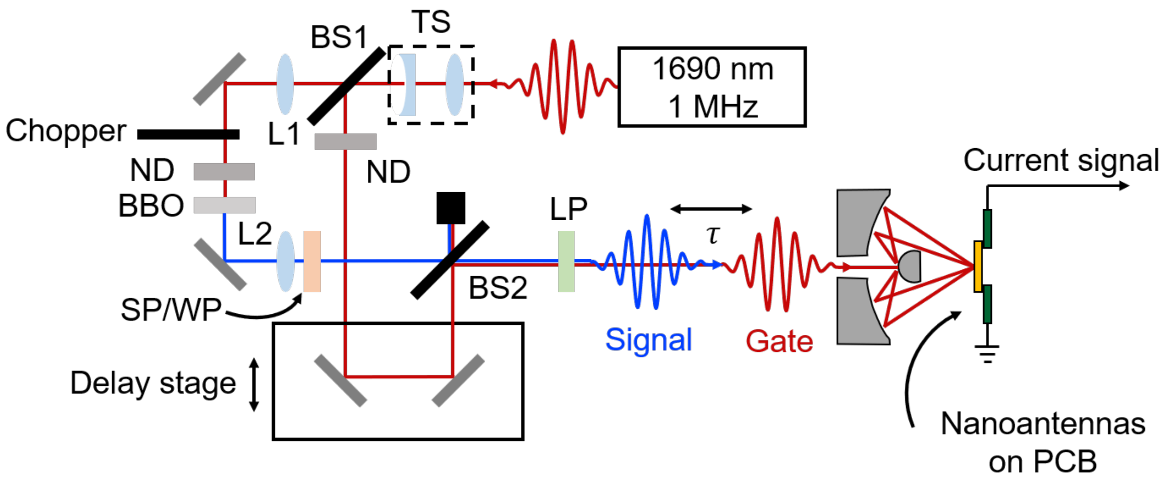

Figure S8: **A schematic of the experimental setup for the measurement of the f_{gate} = 0.177 PHz and f_{signal} = SHG of 0.177 PHz.** The laser light first passes through a telescope and is split using a broadband beamsplitter (BS). One arm has a delay stage and was used as the gate pulse, while the chopped signal arm has a lens to focus onto the nonlinear crystal used to double the frequency and another lens was used to collimate the SHG. To control the SHG power, an ND filter was placed before the BBO and a 0.207 PHz (1450 nm) high-frequency pass filter (ND 2 at 0.177 PHz) was used to attenuate the residual f = 0.177 PHz and a broadband achromatic waveplate (WP) was used to rotate the SHG polarization from vertical to horizontal. The two pulses are recombined using an identical broadband beamsplitter before being sent to a reflective objective where they are focused onto the nanoantenna devices

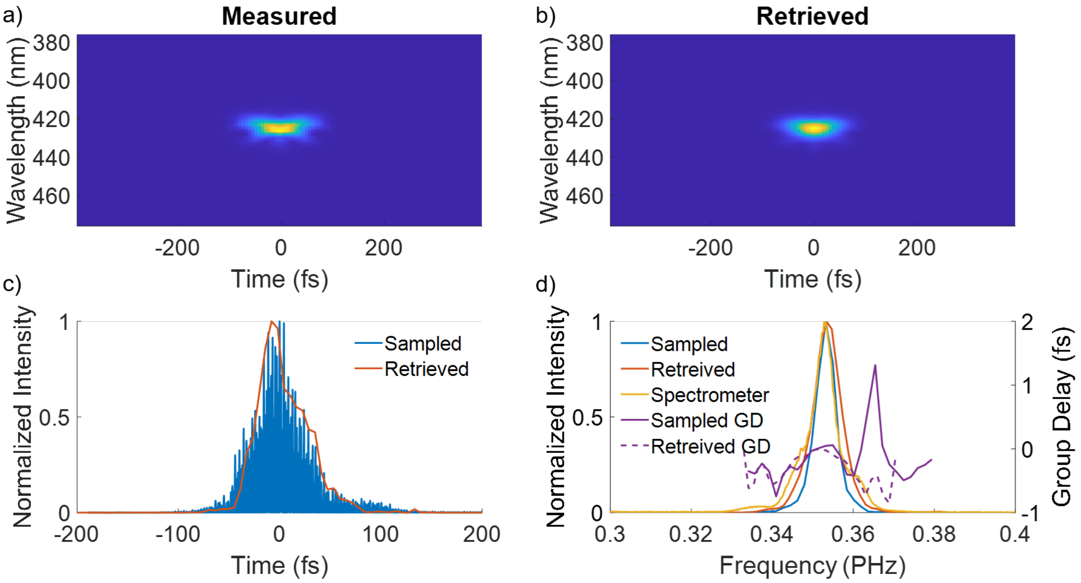

Figure S9: **Frequency-resolved optical gating (FROG) of the $f = 0.353$ PHz pulse.** (a) measured and (b) retrieved spectrograms. (c) A comparison of the squared modulus of sampled optical fields and the retrieved pulse versus time. (d) A comparison of the squared modulus of the Fourier transformed sampled fields, retrieved pulse versus frequency, a spectrometer reference, group delay from the sampled optical fields, and the retrieved group delay.

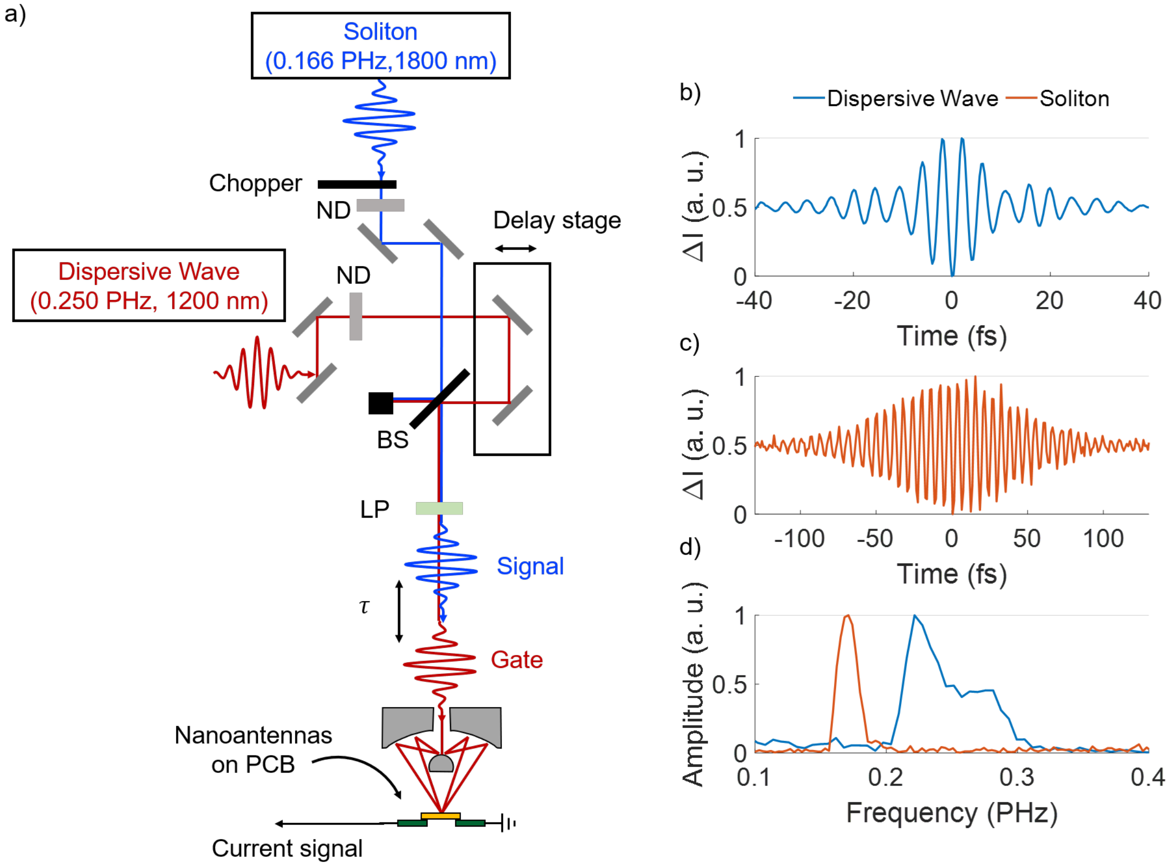

Figure S10: **Downsampling using a supercontinuum pumped by a 78.4 MHz Er Fiber Laser.** (a) The experimental schematic for the measurement. (b) Degenerate sampling of the dispersive wave. (c) Non-degenerate sampling of the soliton using the dispersive wave. (d) The corresponding Fourier transformed sampled fields.

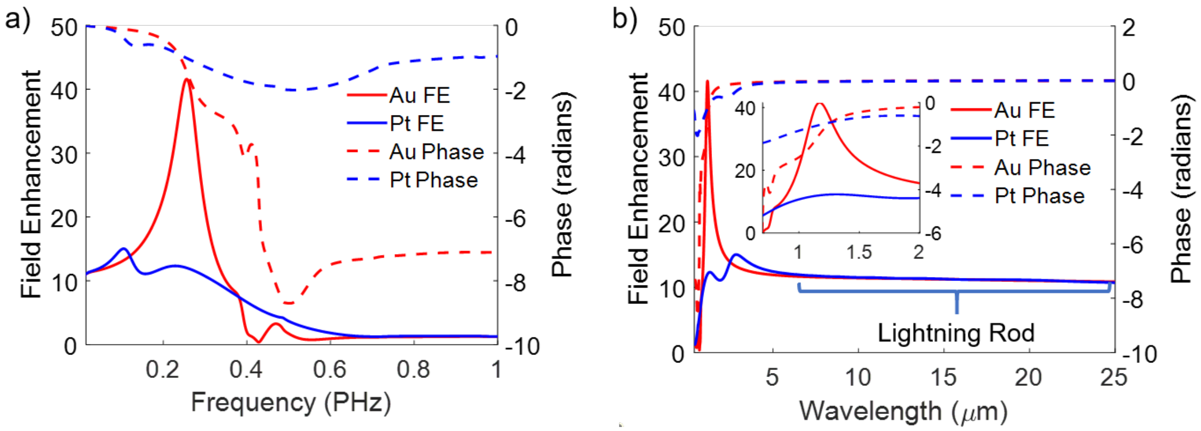

Figure S11: **FDTD simulation of the gold nanoantenna and platinum nanoantenna**. The platinum serves as a reference for a non-plasmonic metal. In (a) is the field enhancement and phase as a function of frequency while (b) is in wavelength

REFERENCES AND NOTES

1. F. Krausz, M. I. Stockman, Attosecond metrology: From electron capture to future signal processing. *Nat. Photonics* **8**, 205–213 (2014).

2. M. Borsch, M. Meierhofer, R. Huber, M. Kira, Lightwave electronics in condensed matter. *Nat. Rev. Mater.* **8**, 668–687 (2023).

3. J. Schoetz, Z. Wang, E. Pisanty, M. Lewenstein, M. F. Kling, M. F. Ciappina, Perspective on petahertz electronics and attosecond nanoscopy. *ACS Photonics* **6**, 3057–3069 (2019).

4. M. F. Ciappina, J. A. Pérez-Hernández, A. S. Landsman, W. A. Okell, S. Zherebtsov, B. Förg, J. Schötz, L. Seiffert, T. Fennel, T. Shaaran, T. Zimmermann, A. Chacón, R. Guichard, A. Zaïr, J. W. G. Tisch, J. P. Marangos, T. Witting, A. Braun, S. A. Maier, L. Roso, M. Krüger, P. Hommelhoff, M. F. Kling, F. Krausz, M. Lewenstein, Attosecond physics at the nanoscale. *Rep. Prog. Phys.* **80**, 054401 (2017).

5. C. Karnetzky, P. Zimmermann, C. Trummer, C. Duque Sierra, M. Wörle, R. Kienberger, A. Holleitner, Towards femtosecond on-chip electronics based on plasmonic hot electron nano-emitters *Communications* **9**, 2471 (2018).

6. K. M. Evenson, G. W. Day, J. S. Wells, L. O. Mullen, Extension of absolute frequency measurements to the cw He–Ne laser at 88 THz (3.39 μ). *Appl. Phys. Lett.* **20**, 133–134 (1972).

7. J. G. Small, G. M. Elchinger, A. Javan, A. Sanchez, F. J. Bachner, D. L. Smythe, Ac electron tunneling at infrared frequencies: Thin-film M-O-M diode structure with broad-band characteristics. *Appl. Phys. Lett.* **24**, 275–279 (1974).

8. T. Rybka, M. Ludwig, M. F. Schmalz, V. Knittel, D. Brida, A. Leitenstorfer, Sub-cycle optical phase control of nanotunnelling in the single-electron regime. *Nat. Photonics* **10**, 667–670 (2016).

9. M. Ludwig, G. Aguirregabiria, F. Ritzkowsky, T. Rybka, D. C. Marinica, J. Aizpurua, A. G. Borisov, A. Leitenstorfer, D. Brida, Sub-femtosecond electron transport in a nanoscale gap. *Nat. Phys.* **16**, 341–345 (2020).

10. W. P. Putnam, R. G. Hobbs, P. D. Keathley, K. K. Berggren, F. X. Kärtner, Optical-field-controlled photoemission from plasmonic nanoparticles. *Nat. Phys.* **13**, 335–339 (2017).

11. P. D. Keathley, W. P. Putnam, P. Vasireddy, R. G. Hobbs, Y. Yang, K. K. Berggren, F. X. Kärtner, Vanishing carrier-envelope-phase-sensitive response in optical-field photoemission from plasmonic nanoantennas. *Nat. Phys.* **15**, 1128–1133 (2019).

12. Y. Yang, M. Turchetti, P. Vasireddy, W. P. Putnam, O. Karnbach, A. Nardi, F. X. Kärtner, K. K. Berggren, P. D. Keathley, Light phase detection with on-chip petahertz electronic networks. *Nat. Commun.* **11**, 3407 (2020).

13. V. Hanus, B. Fehér, V. Csajbók, P. Sándor, Z. Pápa, J. Budai, Z. Wang, P. Paul, A. Szeghalmi, P. Dombi, Carrier-envelope phase on-chip scanner and control of laser beams. *Nat. Commun.* **14**, 5068 (2023).

14. M. R. Bionta, F. Ritzkowsky, M. Turchetti, Y. Yang, D. Cattozzo Mor, W. P. Putnam, F. X. Kärtner, K. K. Berggren, P. D. Keathley, On-chip sampling of optical fields with attosecond resolution. *Nat. Photonics* **15**, 456–460 (2021).

15. J. Blöchl, J. Schötz, A. Maliakkal, N. Šreibere, Z. Wang, P. Rosenberger, P. Hommelhoff, A. Staudte, P. B. Corkum, B. Bergues, M. F. Kling, Spatiotemporal sampling of near-petahertz vortex fields. *Optica* **9**, 755–761 (2022).

16. H. Y. Kim, M. Garg, S. Mandal, L. Seiffert, T. Fennel, E. Goulielmakis, Attosecond field emission. *Nature* **613**, 662–666 (2023).

17. L. Wimmer, G. Herink, D. R. Solli, S. V. Yalunin, K. E. Echternkamp, C. Ropers, Terahertz control of nanotip photoemission. *Nat. Phys.* **10**, 432–436 (2014).

18. P. Dombi, A. Hörl, P. Rácz, I. Márton, A. Trügler, J. R. Krenn, U. Hohenester, Ultrafast strong-field photoemission from plasmonic nanoparticles. *Nano Lett.* **13**, 674–678 (2013).

19. P. Dienstbier, L. Seiffert, T. Paschen, A. Liehl, A. Leitenstorfer, T. Fennel, P. Hommelhoff, Tracing attosecond electron emission from a nanometric metal tip. *Nature* **616**, 702–706 (2023).

20. C. Ropers, D. R. Solli, C. P. Schulz, C. Lienau, T. Elsaesser, Localized multiphoton emission of femtosecond electron pulses from metal nanotips. *Phys. Rev. Lett.* **98**, 043907 (2007).

21. Z. Wang, H. Park, Y. H. Lai, J. Xu, C. I. Blaga, F. Yang, P. Agostini, L. F. DiMauro, The roles of photo-carrier doping and driving wavelength in high harmonic generation from a semiconductor. *Nat. Commun.* **8**, 1686 (2017).

22. P. D. Keathley, S. V. B. Jensen, M. Yeung, M. R. Bionta, L. B. Madsen, Uncovering extreme nonlinear dynamics in solids through time-domain field analysis. *Phys. Rev. B* **107**, 054302 (2023).

23. P.-C. Huang, C. Hernández-García, J.-T. Huang, P.-Y. Huang, C.-H. Lu, L. Rego, D. D. Hickstein, J. L. Ellis, A. Jaron-Becker, A. Becker, S.-D. Yang, C. G. Durfee, L. Plaja, H. C. Kapteyn, M. M. Murnane, A. H. Kung, M.-C. Chen, Polarization control of isolated high-harmonic pulses. *Nat. Photonics* **12**, 349–354 (2018).

24. O. Schubert, M. Hohenleutner, F. Langer, B. Urbanek, C. Lange, U. Huttner, D. Golde, T. Meier, M. Kira, S. W. Koch, R. Huber, Sub-cycle control of terahertz high-harmonic generation by dynamical Bloch oscillations. *Nat. Photonics* **8**, 119–123 (2014).

25. M. Hohenleutner, F. Langer, O. Schubert, M. Knorr, U. Huttner, S. W. Koch, M. Kira, R. Huber, Real-time observation of interfering crystal electrons in high-harmonic generation. *Nature* **523**, 572–575 (2015).

26. S. Ito, M. Schüler, M. Meierhofer, S. Schlauderer, J. Freudenstein, J. Reimann, D. Afanasiev, K. A. Kokh, O. E. Tereshchenko, J. Güdde, M. A. Sentef, U. Höfer, R. Huber, Build-up and dephasing of Floquet–Bloch bands on subcycle timescales. *Nature* **616**, 696–701 (2023).

27. C. Jin, E. Y. Ma, O. Karni, E. C. Regan, F. Wang, T. F. Heinz, Ultrafast dynamics in van der Waals heterostructures. *Nat. Nanotechnol.* **13**, 994–1003 (2018).

28. J. Reimann, S. Schlauderer, C. P. Schmid, F. Langer, S. Baierl, K. A. Kokh, O. E. Tereshchenko, A. Kimura, C. Lange, J. Güdde, U. Höfer, R. Huber, Subcycle observation of lightwave-driven Dirac currents in a topological surface band. *Nature* **562**, 396–400 (2018).

29. C. P. Schmid, L. Weigl, P. Grössing, V. Junk, C. Gorini, S. Schlauderer, S. Ito, M. Meierhofer, N. Hofmann, D. Afanasiev, J. Crewse, K. A. Kokh, O. E. Tereshchenko, J. Güdde, F. Evers, J. Wilhelm, K. Richter, U. Höfer, R. Huber, Tunable non-integer high-harmonic generation in a topological insulator. *Nature* **593**, 385–390 (2021).

30. F. Langer, M. Hohenleutner, U. Huttner, S. W. Koch, M. Kira, R. Huber, Symmetry-controlled temporal structure of high-harmonic carrier fields from a bulk crystal. *Nat. Photonics* **11**, 227–231 (2017).

31. D. L. Marks, S. A. Boppart, Nonlinear interferometric vibrational imaging. *Phys. Rev. Lett.* **92**, 123905 (2004).

32. V. Kumar, A. De la Cadena, A. Perri, F. Preda, N. Coluccelli, G. Cerullo, D. Polli, Invited article: Complex vibrational susceptibility by interferometric Fourier transform stimulated Raman scattering. *APL Photonics* **3**, 092403 (2018).

33. J. Alves, J. Feng, L. Nienhaus, T. W. Schmidt, Challenges, progress and prospects in solid state triplet fusion upconversion. *J. Mater. Chem. C* **10**, 7783–7798 (2022).

34. S. Wen, J. Zhou, P. J. Schuck, Y. D. Suh, T. W. Schmidt, D. Jin, Future and challenges for hybrid upconversion nanosystems. *Nat. Photonics* **13**, 828–838 (2019).

35. T. Wang, C. Xu, Three-photon neuronal imaging in deep mouse brain. *Optica* **7**, 947–960 (2020).

36. C. L. Evans, X. S. Xie, Coherent anti-stokes raman scattering microscopy: Chemical imaging for biology and medicine. *Annu. Rev. Anal. Chem.* **1**, 883–909 (2008).

37. M. Schultze, E. M. Bothschafter, A. Sommer, S. Holzner, W. Schweinberger, M. Fiess, M. Hofstetter, R. Kienberger, V. Apalkov, V. S. Yakovlev, M. I. Stockman, F. Krausz, Controlling dielectrics with the electric field of light. *Nature* **493**, 75–78 (2013).

38. A. Schiffrin, T. Paasch-Colberg, N. Karpowicz, V. Apalkov, D. Gerster, S. Mühlbrandt, M. Korbman, J. Reichert, M. Schultze, S. Holzner, J. V. Barth, R. Kienberger, R. Ernstorfer, V. S. Yakovlev, M. I. Stockman, F. Krausz, Optical-field-induced current in dielectrics. *Nature* **493**, 70–74 (2013).

39. S. Sederberg, D. Zimin, S. Keiber, F. Siegrist, M. S. Wismer, V. S. Yakovlev, I. Floss, C. Lemell, J. Burgdörfer, M. Schultze, F. Krausz, N. Karpowicz, Attosecond optoelectronic field measurement in solids. *Nat. Commun.* **11**, 430 (2020).

40. T. Higuchi, C. Heide, K. Ullmann, H. B. Weber, P. Hommelhoff, Light-field-driven currents in graphene. *Nature* **550**, 224–228 (2017).

41. S. B. Park, K. Kim, W. Cho, S. I. Hwang, I. Ivanov, C. H. Nam, K. T. Kim, Direct sampling of a light wave in air. *Optica* **5**, 402–408 (2018).

42. D. Zimin, M. Weidman, J. Schötz, M. F. Kling, V. S. Yakovlev, F. Krausz, N. Karpowicz, Petahertz-scale nonlinear photoconductive sampling in air. *Optica* **8**, 586–590 (2021).

43. N. Altwaijry, M. Qasim, M. Mamaikin, J. Schötz, K. Golyari, M. Heynck, E. Ridente, V. S. Yakovlev, N. Karpowicz, M. F. Kling, Broadband photoconductive sampling in gallium phosphide. *Adv. Opt. Mater.* **11**, 2202994 (2023).

44. D. Hui, H. Alqattan, S. Yamada, V. Pervak, K. Yabana, M. T. Hassan, Attosecond electron motion control in dielectric. *Nat. Photonics* **16**, 33–37 (2022).

45. K. T. Kim, C. Zhang, A. D. Shiner, B. E. Schmidt, F. Légaré, D. M. Villeneuve, P. B. Corkum, Petahertz optical oscilloscope. *Nat. Photonics* **7**, 958–962 (2013).

46. A. Herbst, K. Scheffter, M. M. Bidhendi, M. Kieker, A. Srivastava, H. Fattahi, Recent advances in petahertz electric field sampling. *J. Phys. B. Atom. Molec. Opt. Phys.* **55**, 172001 (2022).

47. W. Cho, S. I. Hwang, C. H. Nam, M. R. Bionta, P. Lassonde, B. E. Schmidt, H. Ibrahim, F. Légaré, K. T. Kim, Temporal characterization of femtosecond laser pulses using tunneling ionization in the UV, visible, and mid-IR ranges. *Sci. Rep.* **9**, 16067 (2019).

48. Y. Liu, J. E. Beetar, J. Nesper, S. Gholam-Mirzaei, M. Chini, Single-shot measurement of few-cycle optical waveforms on a chip. *Nat. Photonics* **16**, 109–112 (2022).

49. A. Wirth, M. Th. Hassan, I. Grguraš, J. Gagnon, A. Moulet, T. T. Luu, S. Pabst, R. Santra, Z. A. Alahmed, A. M. Azzeer, V. S. Yakovlev, V. Pervak, F. Krausz, E. Goulielmakis, Synthesized light transients. *Science* **334**, 195–200 (2011).

50. R. Borrego-Varillas, M. Lucchini, M. Nisoli, Attosecond spectroscopy for the investigation of ultrafast dynamics in atomic, molecular and solid-state physics. *Rep. Prog. Phys.* **85**, 066401 (2022).

51. F. Ritzkowsky, M. Yeung, E. Bebeti, T. Gebert, T. Matsuyama, M. Budden, R. Mainz, H. Cankaya, K. Berggren, G. Rossi, P. Keathley, F. Kärtner, Large area optical frequency detectors for single-shot phase readout. arXiv:2306.01621 [physics.optics] (2023).

52. F. Ferdous, D. E. Leaird, C.-B. Huang, A. M. Weiner, Dual-comb electric-field cross-correlation technique for optical arbitrary waveform characterization. *Opt. Lett.* **34**, 3875–3877 (2009).

53. F. Ritzkowsky, M. R. Bionta, M. Turchetti, K. K. Berggren, F. X. Kärtner, P. D. Keathley, Engineering the frequency response of petahertz-electronic nanoantenna field-sampling devices, in *Conference on Lasers and Electro-Optics (2022), Paper JW3A.56* (Optica Publishing Group, 2022), p. JW3A.56.

54. K. Arai, D. Okazaki, I. Morichika, S. Ashihara, All-solid-state optical-field-sensitive detector for sub-nanojoule pulses using metal–insulator hybrid nanostructure. *ACS Photonics* **10**, 1702–1707 (2023).

55. D. A. Zimin, V. S. Yakovlev, N. Karpowicz, Ultra-broadband all-optical sampling of optical waveforms *Advances* **8**, eade1029 (2022).

56. M. Mamaikin, Y.-L. Li, E. Ridente, W. T. Chen, J.-S. Park, A. Y. Zhu, F. Capasso, M. Weidman, M. Schultze, F. Krausz, N. Karpowicz, Electric-field-resolved near-infrared microscopy. *Optica* **9**, 616–622 (2022).

57. E. Ridente, M. Mamaikin, N. Altwaijry, D. Zimin, M. F. Kling, V. Pervak, M. Weidman, F. Krausz, N. Karpowicz, Electro-optic characterization of synthesized infrared-visible light fields. *Nat. Commun.* **13**, 1111 (2022).

58. S. Keiber, S. Sederberg, A. Schwarz, M. Trubetskov, V. Pervak, F. Krausz, N. Karpowicz, Electro-optic sampling of near-infrared waveforms. *Nat. Photonics* **10**, 159–162 (2016).

59. P. Sulzer, K. Oguchi, J. Huster, M. Kizmann, T. L. M. Guedes, A. Liehl, C. Beckh, A. S. Moskalenko, G. Burkard, D. V. Seletskiy, A. Leitenstorfer, Determination of the electric field and its Hilbert transform in femtosecond electro-optic sampling. *Phys. Rev. A* **101**, 033821 (2020).

60. I. Pupeza, M. Huber, M. Trubetskov, W. Schweinberger, S. A. Hussain, C. Hofer, K. Fritsch, M. Poetzlberger, L. Vamos, E. Fill, T. Amotchkina, K. V. Kepesidis, A. Apolonski, N. Karpowicz, V. Pervak, O. Pronin, F. Fleischmann, A. Azzeer, M. Žigman, F. Krausz, Field-resolved infrared spectroscopy of biological systems. *Nature* **577**, 52–59 (2020).

61. P. Steinleitner, N. Nagl, M. Kowalczyk, J. Zhang, V. Pervak, C. Hofer, A. Hudzikowski, J. Sotor, A. Weigel, F. Krausz, K. F. Mak, Single-cycle infrared waveform control. *Nat. Photonics* **16**, 512–518 (2022).

62. D. M. B. Lesko, H. Timmers, S. Xing, A. Kowligy, A. J. Lind, S. A. Diddams, A six-octave optical frequency comb from a scalable few-cycle erbium fibre laser. *Nat. Photonics* **15**, 281–286 (2021).

63. E. Fresch, F. V. A. Camargo, Q. Shen, C. C. Bellora, T. Pullerits, G. S. Engel, G. Cerullo, E. Collini, Two-dimensional electronic spectroscopy. *Nat. Rev. Meth. Primers* **3**, 1–16 (2023).

64. R. Jafari, T. Jones, R. Trebino, 100% Reliable algorithm for second-harmonic-generation frequency-resolved optical gating. *Opt. Express* **27**, 2112–2124 (2019).

65. A. F. Oskooi, D. Roundy, M. Ibanescu, P. Bermel, J. D. Joannopoulos, S. G. Johnson, Meep: A flexible free-software package for electromagnetic simulations by the FDTD method. *Comput. Phys. Commun.* **181**, 687–702 (2010).

66. R. Bhattacharya, M. Turchetti, M. Yeung, P. Donald Keathley, K. K. Berggren, J. Browning, Effect of ultraviolet light on field emission performance and lifetime of lateral field emitter devices. *J. Vac. Sci. Technol. B* **41**, 063202 (2023).

67. W. P. Putnam, P. D. Keathley, J. A. Cox, A. Liehl, A. Leitenstorfer, F. X. Kärtner, Few-cycle, carrier–envelope-phase-stable laser pulses from a compact supercontinuum source. *JOSA B* **36**, A93–A97 (2019).
